# Supplementary material for: Incisor Occlusion Affects Profile Shape Variation in Middle-Aged Adults
Source: J Clin Med. 2021 Feb 17;10(4):800. doi: 10.3390/jcm10040800 (PMC7923002; doi:10.3390/jcm10040800)
Supplement: Supplementary file 1 [file jcm-10-00800-s001.pdf]

Supplementary Material

Incisor occlusion affects profile shape variation in middle-aged adults

Georgios Kanavakis [1,2,3], Anna-Sofia Silvola [3,4], Demetrios Halazonetis [5], Raija Lähdesmäki [3,4], Pertti Pirttiniemi [3,4]

1 Department of Pediatric Oral Health and Orthodontics, UZB-University Center for Dental Medicine, University of Basel, Switzerland
2 Department of Orthodontics and Dentofacial Orthopedics, Tufts University School of Dental Medicine, Boston MA, USA
3 Oral Development and Orthodontics, Research Unit of Oral Health Sciences, Medical Faculty, University of Oulu, Finland
4 Oral and Maxillofacial Department, Oulu University Hospital, Medical Research Center Oulu (MRC Oulu)
5 Department of Orthodontics, School of Dentistry, National and Kapodistrian University of Athens, Greece

*Correspondence:
Georgios Kanavakis
Department of Pediatric Oral Health and Orthodontics
University Center for Dental Medicine - UZB
Mattenstrasse 40
CH-4058 Basel
Tel: +41 (0) 61 267 26 15
Fax: +41 (0) 61 267 25 81
Email: georgios.kanavakis@unibas.ch; gkanavak@gmail.com

Table S1: Comparison between males and females in overjet and overbite.

		N	Mean	SD	P-value
Overjet	Males	799	2.90	1.90	< 0.001
	Females	955	3.25	1.91	
Overbite	Males	799	3.41	2.07	0.971
	Females	955	3.41	1.67	

Table S2: Between-subjects effect of overjet and overbite on profile shape variation, as explained by PC1-PC9.

Profile shape PCs	Variation explained (%)		η^2	P-value
PC1	33.1	Overjet	0.070	<0.001
		Overbite	0.013	<0.001
PC2	23.1	Overjet	0.004	0.013
		Overbite	0.035	<0.001
PC3	11	Overjet	0.006	0.002
		Overbite	0.001	0.246
PC4	6.7	Overjet	0.049	<0.001
		Overbite	0.041	<0.001
PC5	5.1	Overjet	0.000	0.620
		Overbite	0.004	0.011
PC6	4	Overjet	0.012	<0.001
		Overbite	0.007	0.001
PC7	3.2	Overjet	0.040	<0.001
		Overbite	0.015	<0.001
PC8	3.1	Overjet	0.002	0.057
		Overbite	0.003	0.032
PC9	2.2	Overjet	0.018	<0.001
		Overbite	0.014	<0.001

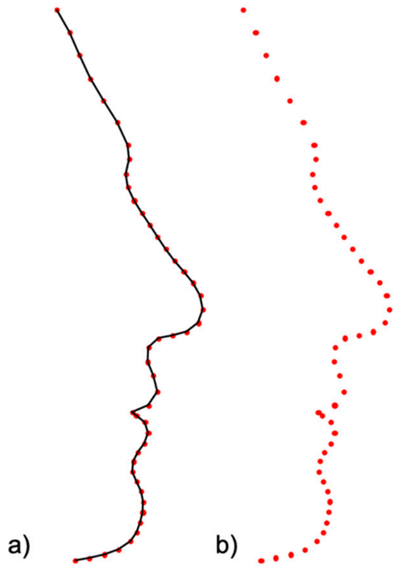

Figure S1: Schematic of the digitization process. The image on the left (a) depicts the upper and lower facial curvatures (black lines) that were placed first, along each profile image. The red dots represent the landmarks that were then positioned along the curves either manually (fixed landmarks) or automatically (semi-landmarks). The image on the right (b) depicts the landmark configuration that was used for all shape analyses as description of facial profile.

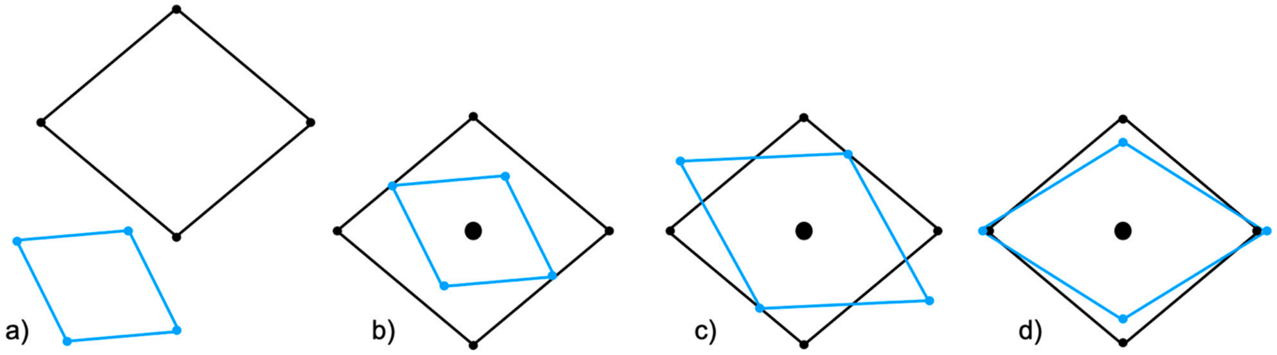

Figure S2: Procrustes Superimposition. To compare the shapes of the black and the blue landmark configurations (a), those are first centered to a common centroid (b). The next step is to scale the two configurations to a fixed centroid size in order to eliminate differences in size. Finally, the two landmark configurations are rotated so that the sum of squared distances between corresponding landmarks is minimized (d). Following these steps, pure shape comparisons between various landmark configurations are enabled.
Note: This schematic is purely descriptive and is meant to support readers' understanding of Procrustes Superimposition.
